# Supplementary material for: Unveiling the Bioactive Potential of the Invasive Jellyfish Phyllorhiza punctata Through Integrative Transcriptomic and Proteomic Analyses
Source: Biomolecules. 2025 Aug 4;15(8):1121. doi: 10.3390/biom15081121 (PMC12383608; doi:10.3390/biom15081121)
Supplement: Supplementary file 1 [file biomolecules-15-01121-s001.zip › Suplementary Material/FileS3/PEPFOLD4/XP_065053997_12370/clusters.html]

 PEP-FOLD clusters


Download archive of all models  here   

|  |  |  |
| --- | --- | --- |
| # | file | sOPEP |
| # cluster 1 - Size: 31 |  |  |
|  | XP\_065053997\_12370-model1 | -11.9267 |
|  | XP\_065053997\_12370-model1.1 | -11.8706 |
|  | XP\_065053997\_12370-model1.2 | -11.8706 |
|  | XP\_065053997\_12370-model1.3 | -11.8554 |
|  | XP\_065053997\_12370-model1.4 | -11.8051 |
|  | XP\_065053997\_12370-model1.5 | -11.7945 |
|  | XP\_065053997\_12370-model1.6 | -11.697 |
|  | XP\_065053997\_12370-model1.7 | -11.697 |
|  | XP\_065053997\_12370-model1.8 | -11.6307 |
|  | XP\_065053997\_12370-model1.9 | -11.6307 |
|  | XP\_065053997\_12370-model1.10 | -11.6307 |
|  | XP\_065053997\_12370-model1.11 | -11.6307 |
|  | XP\_065053997\_12370-model1.12 | -11.6307 |
|  | XP\_065053997\_12370-model1.13 | -11.6307 |
|  | XP\_065053997\_12370-model1.14 | -11.6307 |
|  | XP\_065053997\_12370-model1.15 | -11.6307 |
|  | XP\_065053997\_12370-model1.16 | -11.6307 |
|  | XP\_065053997\_12370-model1.17 | -11.6057 |
|  | XP\_065053997\_12370-model1.18 | -11.6057 |
|  | XP\_065053997\_12370-model1.19 | -11.6057 |
|  | XP\_065053997\_12370-model1.20 | -11.6057 |
|  | XP\_065053997\_12370-model1.21 | -11.6057 |
|  | XP\_065053997\_12370-model1.22 | -11.6057 |
|  | XP\_065053997\_12370-model1.23 | -11.6057 |
|  | XP\_065053997\_12370-model1.24 | -11.6057 |
|  | XP\_065053997\_12370-model1.25 | -11.6057 |
|  | XP\_065053997\_12370-model1.26 | -11.5836 |
|  | XP\_065053997\_12370-model1.27 | -11.4867 |
|  | XP\_065053997\_12370-model1.28 | -11.4692 |
|  | XP\_065053997\_12370-model1.29 | -11.211 |
|  | XP\_065053997\_12370-model1.30 | -11.211 |
| # cluster 2 - Size: 41 |  |  |
|  | XP\_065053997\_12370-model2 | -7.77236 |
|  | XP\_065053997\_12370-model2.1 | -7.72728 |
|  | XP\_065053997\_12370-model2.2 | -7.72728 |
|  | XP\_065053997\_12370-model2.3 | -7.72728 |
|  | XP\_065053997\_12370-model2.4 | -7.68904 |
|  | XP\_065053997\_12370-model2.5 | -7.65133 |
|  | XP\_065053997\_12370-model2.6 | -7.65133 |
|  | XP\_065053997\_12370-model2.7 | -7.65133 |
|  | XP\_065053997\_12370-model2.8 | -7.65133 |
|  | XP\_065053997\_12370-model2.9 | -7.65133 |
|  | XP\_065053997\_12370-model2.10 | -7.65133 |
|  | XP\_065053997\_12370-model2.11 | -7.64545 |
|  | XP\_065053997\_12370-model2.12 | -7.63313 |
|  | XP\_065053997\_12370-model2.13 | -7.61082 |
|  | XP\_065053997\_12370-model2.14 | -7.61082 |
|  | XP\_065053997\_12370-model2.15 | -7.60672 |
|  | XP\_065053997\_12370-model2.16 | -7.60672 |
|  | XP\_065053997\_12370-model2.17 | -7.57048 |
|  | XP\_065053997\_12370-model2.18 | -7.57048 |
|  | XP\_065053997\_12370-model2.19 | -7.55683 |
|  | XP\_065053997\_12370-model2.20 | -7.53231 |
|  | XP\_065053997\_12370-model2.21 | -7.53231 |
|  | XP\_065053997\_12370-model2.22 | -7.53199 |
|  | XP\_065053997\_12370-model2.23 | -7.51937 |
|  | XP\_065053997\_12370-model2.24 | -7.50856 |
|  | XP\_065053997\_12370-model2.25 | -7.49593 |
|  | XP\_065053997\_12370-model2.26 | -7.49593 |
|  | XP\_065053997\_12370-model2.27 | -7.49593 |
|  | XP\_065053997\_12370-model2.28 | -7.49593 |
|  | XP\_065053997\_12370-model2.29 | -7.49593 |
|  | XP\_065053997\_12370-model2.30 | -7.49593 |
|  | XP\_065053997\_12370-model2.31 | -7.48357 |
|  | XP\_065053997\_12370-model2.32 | -7.48357 |
|  | XP\_065053997\_12370-model2.33 | -7.48337 |
|  | XP\_065053997\_12370-model2.34 | -7.48337 |
|  | XP\_065053997\_12370-model2.35 | -7.48337 |
|  | XP\_065053997\_12370-model2.36 | -7.46736 |
|  | XP\_065053997\_12370-model2.37 | -7.46736 |
|  | XP\_065053997\_12370-model2.38 | -7.44563 |
|  | XP\_065053997\_12370-model2.39 | -7.40091 |
|  | XP\_065053997\_12370-model2.40 | -7.27811 |
| # cluster 3 - Size: 21 |  |  |
|  | XP\_065053997\_12370-model3 | -7.74876 |
|  | XP\_065053997\_12370-model3.1 | -7.74876 |
|  | XP\_065053997\_12370-model3.2 | -7.64923 |
|  | XP\_065053997\_12370-model3.3 | -7.62699 |
|  | XP\_065053997\_12370-model3.4 | -7.62699 |
|  | XP\_065053997\_12370-model3.5 | -7.62699 |
|  | XP\_065053997\_12370-model3.6 | -7.55087 |
|  | XP\_065053997\_12370-model3.7 | -7.55087 |
|  | XP\_065053997\_12370-model3.8 | -7.53938 |
|  | XP\_065053997\_12370-model3.9 | -7.522 |
|  | XP\_065053997\_12370-model3.10 | -7.522 |
|  | XP\_065053997\_12370-model3.11 | -7.52141 |
|  | XP\_065053997\_12370-model3.12 | -7.50946 |
|  | XP\_065053997\_12370-model3.13 | -7.50946 |
|  | XP\_065053997\_12370-model3.14 | -7.50294 |
|  | XP\_065053997\_12370-model3.15 | -7.49924 |
|  | XP\_065053997\_12370-model3.16 | -7.4875 |
|  | XP\_065053997\_12370-model3.17 | -7.46048 |
|  | XP\_065053997\_12370-model3.18 | -7.43097 |
|  | XP\_065053997\_12370-model3.19 | -7.31449 |
|  | XP\_065053997\_12370-model3.20 | -7.31449 |
| # cluster 4 - Size: 5 |  |  |
|  | XP\_065053997\_12370-model4 | -7.55514 |
|  | XP\_065053997\_12370-model4.1 | -7.55373 |
|  | XP\_065053997\_12370-model4.2 | -7.32479 |
|  | XP\_065053997\_12370-model4.3 | -7.32479 |
|  | XP\_065053997\_12370-model4.4 | -7.32479 |
| # cluster 5 - Size: 2 |  |  |
|  | XP\_065053997\_12370-model5 | -7.39311 |
|  | XP\_065053997\_12370-model5.1 | -7.30621 |
|  |  |  |
